# English Quarantine

**Published:** 1879-01

**Authors:** 


### English Quarantine.

Medical Times and Gazette: It is satisfactory to find that the government has not been unmindful of the possible consequences of the present epidemic of yellow fever in America, but has issued the following circular through the local government board to the port sanitary authorities: "I am directed by the president of the local government board to state that, in view of the epidemic of yellow fever which is now prevailing in certain parts of America, it appears to him desirable to draw the attention of port sanitary authorities in England and Wales to the possibility of the importation into this country of cases of this fever. Such experience as has hitherto been had in Europe of yellow fever, which is essentially a tropical disease, has shown that it does not spread except under very special conditions. On the rare occasions when it has extended from vessels arriving in European ports, its extension has appeared to be exclusively among persons on shipboard, or employed in the harbor, or living in the immediate vicinity; and persons who have been taken inland sick of the disease have not communicated it to others. The present outbreak, however, appears to be of so malignant a character that no care should be wanting on the part of port sanitary authorities to meet the possible contingency of the introduction into this country of a malady so fatal. In England, besides other arrangements of the nature of quarantine that are made in respect of this disease, measures of isolation and disinfection of vessels infected with yellow fever are adopted by the commissioners of customs, under the orders of the privy council, as a part of their duties under quarantine acts. Port sanitary authorities, though not themselves charged under the quarantine acts with the duty of securing this isolation and disinfection, can, on occasion, render important service to the commissions of customs in executing these functions in regard to yellow fever ships. In the event of any case of yellow fever finding its way, notwithstanding the precautionary measures taken by the customs, into any English port, the action of the port sanitary authority should be the same as in the case of common infectious diseases of the country, viz: removal of the patient with all necessary precautions into a place of isolation, and the destruction or disinfection of any articles of clothing or bedding that may have been used by him. The board trusts that the port sanitary authority have already provided themselves with means of such isolation and disinfection; but if the authority have not already done so, the board would take the present occasion of again pressing upon them the importance of having such means at all times in readiness."—*Medical News.*

